# Plasma Deposited Polyoxazoline Films Integration Into Spiral Microfluidics for the Targeted Capture of Size Selected Cells

**DOI:** 10.3389/fchem.2021.690781

**Published:** 2021-05-20

**Authors:** Alexandru A. Gheorghiu, Ines Muguet, James Chakiris, Kit Man Chan, Craig Priest, Melanie Macgregor

**Affiliations:** ^1^ Future Industries Institute, University of South Australia, Adelaide, SA, Australia; ^2^ École Nationale Supérieure de Chimie, de Biologie et de Physique de Bordeaux, Bordeaux INP, Pessac, France

**Keywords:** polyoxazolines, plasma polymers, selective cell capture, microfluidic systems, plasma bonding, diagnostic devices

## Abstract

Biomolecules readily and irreversibly bind to plasma deposited Polyoxazoline thin films in physiological conditions. The unique reactivity of these thin films toward antibodies is driving the development of immunosensing platforms for applications in cancer diagnostics. However, in order for these coatings to be used as advanced immunosensors, they need to be incorporated into microfluidic devices that are sealed via plasma bonding. In this work, the thickness, chemistry and reactivity of the polyoxazoline films were assessed following plasma activation. Films deposited from methyl and isopropenyl oxazoline precursors were integrated into spiral microfluidic devices and biofunctionalized with prostate cancer specific antibodies. Using microbeads as model particles, the design of the spiral microfluidic was optimised to enable the size-based isolation of cancer cells. The device was tested with a mixed cell suspension of healthy and malignant prostate cells. The results showed that, following size-specific separation in the spiral, selective capture was achieved on the immunofunctionalised PPOx surface. This proof of concept study demonstrates that plasma deposited polyoxazoline can be used for immunosensing in plasma bonded microfluidic devices.

## Introduction

Plasma deposited Polyoxazoline (PPOx) are a new class of functional coatings which have been developed for use in immunosensors for their unique ability to irreversibly bind antibodies, in a one-step click-chemistry type of reaction in physiological conditions (Macgregor and Vasilev, 2019). The plasma polymer deposition process consists of igniting the vapor phase of an organic monomer into a plasma phase rich in ions, radicals, electrons, photons, etc (Yasuda, 2012). Upon interaction with solid surfaces, these reactive species rearrange themselves randomly into a crosslinked pinhole-free film. Nanothin coatings are typically deposited within minutes in mild vacuum conditions (i.e. 10^-1^ mbar). The method is particularly well suited for translational research activities because it is solvent free, substrate independent and scalable. In the biomedical field, commercial examples of plasma polymers-based technologies include PureCoat™ used to enhance cell adhesion in cell culture plasticware, or Tangible™ Hydra PEG for the lubrication of contact lenses. While a range of biocompatible plasma polymers have been identified, new functional coatings that offer both biocompatibility and reactivity toward biomolecules are needed for application in speciality biosensing products.

**FIGURE 1:**
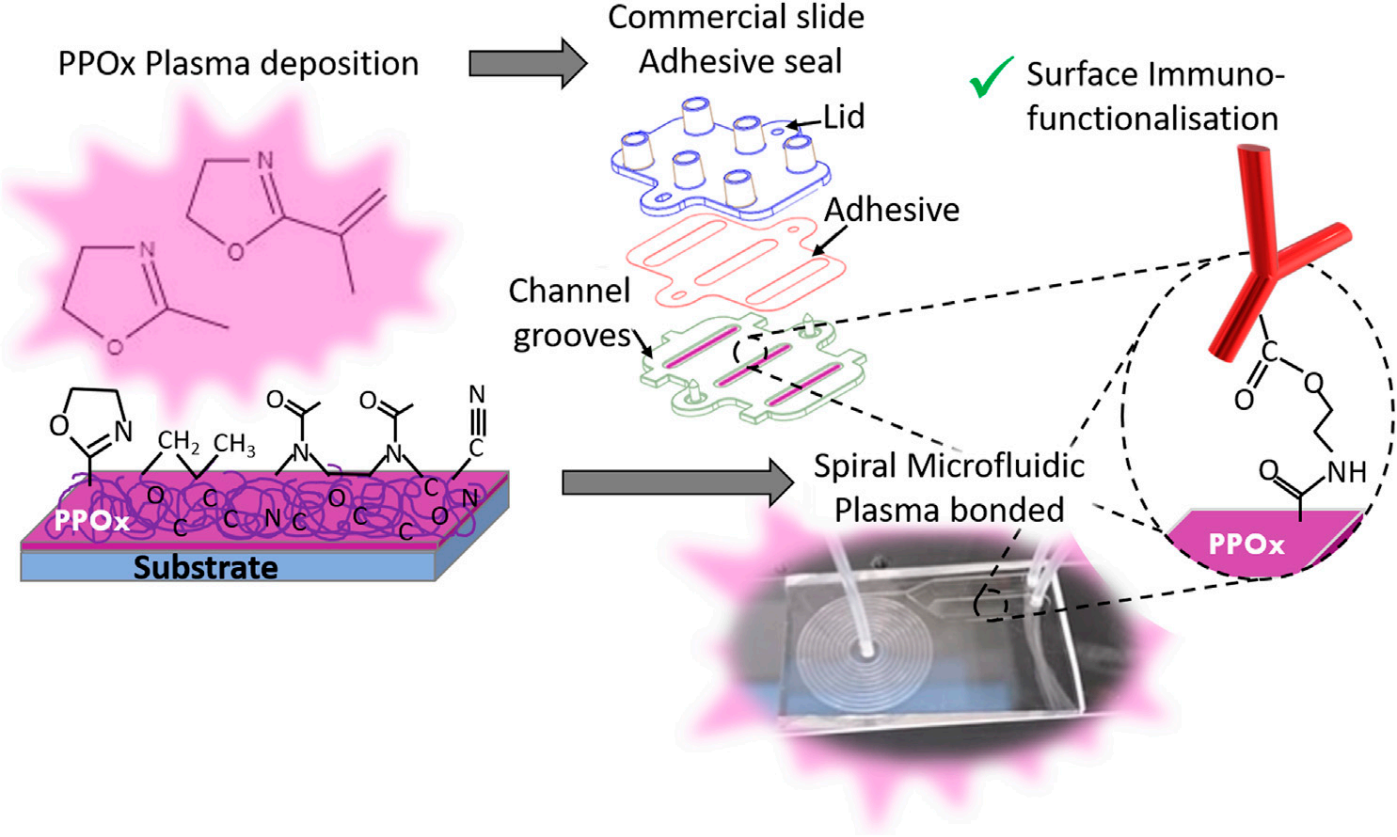
Schematic of the biosensor preparation, showing the PPOx deposition step followed by the chip assembly -via adhesive seal for the commercial slide **(top)** or plasma bonding for the spiral microfluidics **(bottom)**- and the PPOx surface immune-functionalisation. This occurs via formation of an amide ester bond between the oxazoline ring and the carboxylic acid function of the biomolecule.

First reported in 2015, PPOx thin films can be generated from the plasma phase of methyl, ethyl or isopropenyl oxazoline monomers (Bhatt et al., 2015; Ramiasa et al., 2015; Zanini et al., 2018). These starting monomers all contain an oxazoline ring, comprising of three carbons, an oxygen and a nitrogen atom (Figure 1). Following plasma deposition, the thin films exhibit a variety of chemical functionalities including, amine, amide, imine, nitrile and carboxyl functions in addition to intact oxazoline groups. While some surface groups (amides) are common between PPOx and conventional polyoxazolines generated via ring opening polymerisation, others are intrinsic to the plasma deposition process itself (Macgregor et al., 2017). Previous works suggest that plasma-specific reactive groups present on the surface of PPOx films, in particular the oxazoline ring itself, facilitate covalent bonding with molecules or ligands featuring carboxylic acid functionalities (Macgregor-Ramiasa et al., 2015; Zanini et al., 2016). Notably, biomolecules such as proteins and antibodies irreversibly bind to PPOx in PBS (Gonzalez Garcia et al., 2017). Building on this discovery, immunosensing substrates were developed using PPOx to immobilise cancer specific antibodies for application in non-invasive diagnostic devices. The biocompatibility of the films has been previously investigated using both non-cancerous and cancerous prostatic cell lines, fibroblasts, and bladder carcinoma cell lines (Chan K. M. et al., 2020). Cell viability was determined through a resazurin assay and the results showed that PPOx films are biocompatible.

In previous work, PPOx coatings have been used in commercial microfluidic chips for selective isolation of both bladder and prostate cancer cells from urine samples (Macgregor-Ramiasa et al., 2017; Hanieh Safizadeh Shirazi, 2020; Macgregor et al., 2021). These urothelial cancers readily shed cancer cells in urine, so detecting these cancer cells from urine samples could become an alternative to the current invasive diagnostic methods which consist of cystoscopy and/or biopsy (Chan et al., 2020). Urine samples can also be investigated for biomarkers such as exosomes. These extracellular vesicles are more abundant and secreted into biofluids at higher concentrations than the cells they originate from (De Vrij et al., 2013). Exosomes are a useful biomarker as they contain a genomic and proteomic snapshot of their cell of origin (Raposo and Stoorvogel, 2013). In the commercial microfluidic chips used thus far, a simple rectangular groove was first coated with the PPOx thin film before closing the microchannel by gluing the base and lid part together using double sided tape, (Figure 1). Once closed the microchannels were biofunctionalized with the anti-body of choice and blocked with skim milk before loading the cell suspension or patient urine samples, as previously described (Macgregor-Ramiasa et al., 2017; Macgregor et al., 2021). While the devices yield very high sensitivity and specificity with mixed cultured cell suspensions, results were not as conclusive with patient urine samples (Macgregor et al., 2021). The limited performance was attributed to the relatively low numbers of cancer cells shed in urine comparatively to the many other types of background cells (umbrella cells, normal urothelial etc) and smaller metabolites present (Macgregor et al., 2021). From these findings, it transpires that for PPOx to be used in immunosensing, it will need to be integrated into microfluidic chips featuring more intricate designs. That is, systems that add multiple degrees of cancer cells pre-selection based on size, deformability or even permeability criteria using microfeatures such as pillar, contraction-expansion arrays, and other complex microchannel geometries (Li et al., 2007; Wang et al., 2013; Warkiani et al., 2014; Liu et al., 2019). Such chips designs have been reported for the isolation of cancer cells from blood. For instance, spiral microfluidic devices have demonstrated good potential for the size selection of cancer cells amongst red and white blood cells (Wu et al., 2012). In urine, spiral microfluidics could potentially be used to separate cellular materials from other smaller cancer biomarkers such as exosomes, and proteins.

**FIGURE 2:**
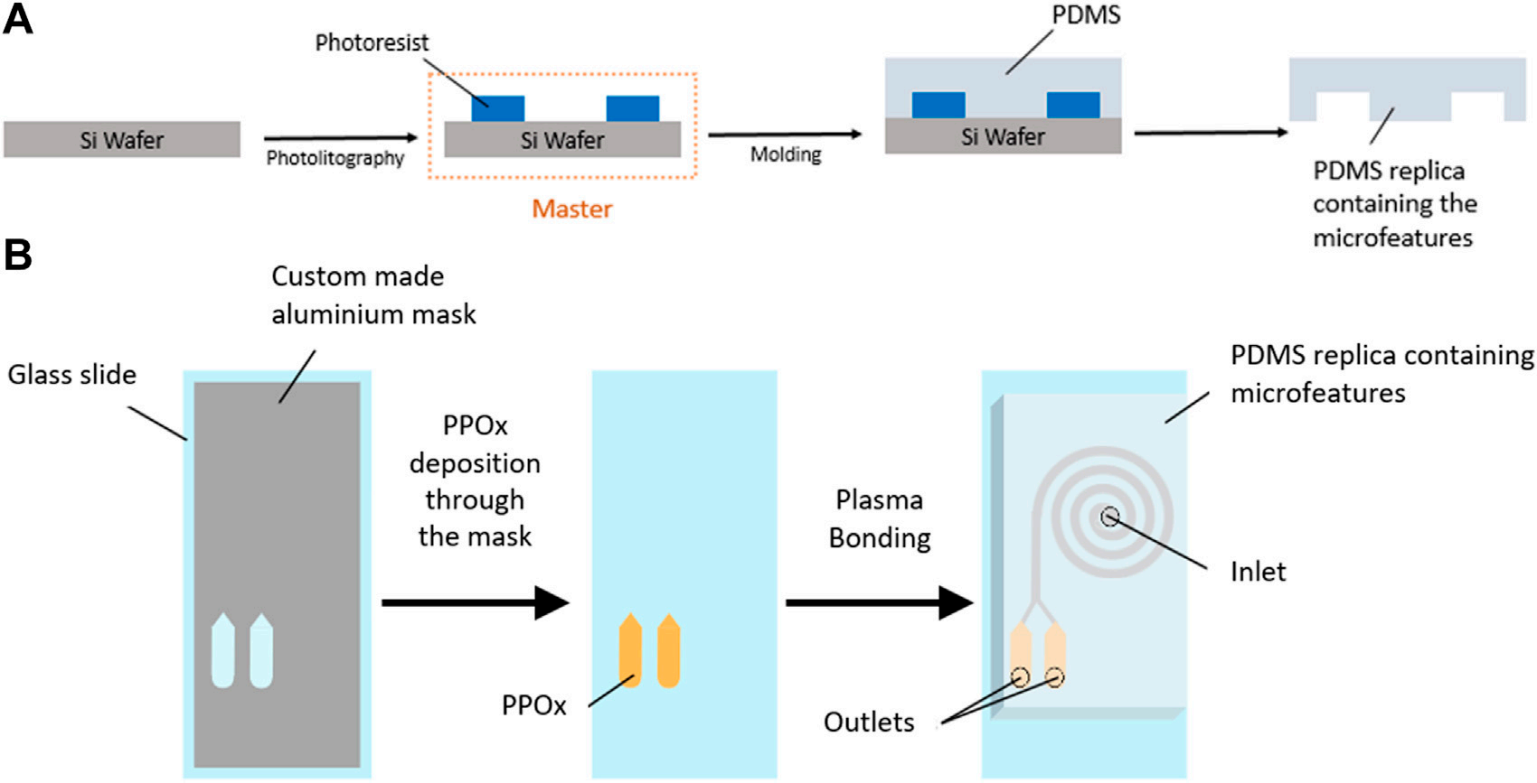
Schematic diagram of SU-8 master fabrication and PDMS molding **(A)** and PPOx deposition and device assembly **(B)**.

These types of microfluidic chips are normally built by bonding micro engineered PDMS to glass, a process requiring plasma activation (Figure 2). During plasma activation, high-energy species from the plasma impact the surface and react with it to generate oxygen containing polar groups that undergo condensation reactions to form Si-O-Si bonds (Bhattacharya et al., 2005). While the plasma polymer deposition approach is well suited to the challenge of coating intricate microfluidic channels shapes, the PPOx film reactivity could be negatively affected by the plasma activation step. In this work we investigated the chemistry and functionality of PPOx films deposited from both methyl and isopropenyl oxazoline following oxygen and air plasma activation. The reactivity of the films post activation was first tested using gold nanoparticles functionalised with mercaptosuccinic acid a model COOH-bearing ligand. The most functional PPOx films were then integrated into a spiral microfluidic chip and biofunctionalized with anti-PSMA antibody to form an immunosensing platform. The spiral microfluidic chip was here used as a model of advanced microfluidic chips capable to isolate cancer cells from body fluids using centrifugal forces. Lastly, we tested the ability of the PPOx-based immunosensor at selectively binding cancer cells following size selection in a spiral microfluidic device.

## Materials and Methods

### Plasma Deposition

Deposition of plasma polymers was carried out in a custom-made reactor that has been previously described previously (Chan K. M. et al., 2020). The plasma was ignited with a 13.56 MHz Radio Frequency generator coupled to a matching network (Coaxial Power System). Substrates, including glass microscope slides, silicon wafers and coverslips, were introduced into the reactor and primed with an air plasma (30 W, 3 min, 0.12 mbar). The reactor was evacuated to a base pressure of 2 × 10^−2^ mbar to remove atmospheric oxygen and nitrogen from the chamber. The pressure was monitored with a pirani gauge (APG100, Edwards) and controlled with a needle valve (Chell Instrument, United Kingdom). The precursors, 2-methyl-2-oxazoline and 2-isopropenyl-2-oxazoline (Sigma Aldrich, Australia) were introduced into the reactor at a working pressure of 0.12 mbar at which point the plasma was ignited at 30 W. Deposition was conducted for 30 and 50 s after which the monomer flow was stopped using the needle valve.

For integration into the spiral microfluidic, PPOx coatings were deposited on glass microscope slides (Marienfeld, Germany) through a custom-made aluminum mask to limit the film deposition to the cell capture reservoir area.

### Thin Film Characterization

Plasma polymer films thickness and chemistry were investigated by ellipsometry, X-ray photoelectron spectroscopy, and time-of-flight secondary ion mass spectrometry. Film thickness measurements were acquired using a spectroscopic ellipsometer (J.A. Woollam, MC-200) from clean silicon wafers with plasma polymer deposited. Data was collected over a wavelength range of 400–800 nm at 65° and 75° whereby thickness was determined using an established Cauchy model and fitting procedure. X-ray photoelectron spectroscopy (XPS) analysis was conducted on a Kratos Axis Ultra DLD spectrometer using a monochromatic Al Ka X-ray source operating at 225 W, corresponding to an energy of 1,486.6 eV. The area of analysis was 0.3. × 0.7 mm and an internal flood gun was employed to minimise charging of the samples. Survey spectra were collected at a dwell time of 55 ms with 160 eV pass energy at steps of 0.5 eV with three sweeps. The data were processed using CasaXPS (Ver. 2.3.16 Casa Software Ltd.). Time-of-Flight Secondary Ion Mass Spectrometry (ToF-SIMS) analysis was carried out using a PHI TRIFT V nanoTOF instrument (Physical Electronics, Inc.) with a pulsed liquid 79+ Au primary ion gun, operated at 30 kV. Dual charge neutralisation was in place in form of an electron flood gun and Ar^+^ ions (both 10 eV). Samples were mounted by gently pressing particles into indium foil, used to avoid charging effects. Collection time per spot was 3 min and the raster size was 10^−4^ cm^2^. All spectra were processed using WincadenceN1 software (Ver. 1.8.1.3 Physical Electronics, Inc.) and further interrogated using NESAC/BIO ToolBox developed at the University of Washington by Dan Graham PhD.

### Thin Film Reactivity Investigation

The reactivity of the POx films toward COOH groups was evaluated by measuring the spontaneous binding of 68 nm gold nanoparticles (AuNPs) capped with mercaptosuccinic acid. The AuNPs were synthesised following well established protocols (Taheri et al., 2018; Visalakshan et al., 2018; Visalakshan et al., 2019). Briefly, 50 ml of 0.01% HAuCl_4_ was boiled and 300 µl of 1% trisodium citrate was added under vigorous stirring. The solution of 68 nm AuNPs was boiled for a further 20 min before the addition of NaOH and mercaptosuccinic acid for surface functionalization with COOH groups. PPOx coated 13 mm glass coverslips were incubated with 400 µl of AuNP solution for 3.5 h in 24 well plates. The AuNP solution was gently aspirated and coverslips rinsed with MilliQ water and dried with nitrogen in preparation for XPS analysis.

### PDMS Chip Fabrication and Plasma Activation

The spiral microchannels incorporating reservoirs at the outlets were designed using AutoCAD 2020 (Autodesk) (see Supplementary Figure SI1). All spirals designs featured one inlet and two outlets downstream from an inner and an outer capture reservoir, and an even bifurcation (dimensions 15 × 3 × 0.17 mm). Spiral microchannels with a rectangular cross section (500 µm wide, 170 µm height) and 2–12 loops were prepared following established procedures. Briefly, SU-8 masters were fabricated by photolithography using a Dilase 650 laser writer lithography system (Kloe, France) (Figure 2). PDMS (Sylgard 184, Sigma-Aldrich, 10:1 ratio of base to curing agent) was casted on the master, degassed and cured at 65°C for 4 h to form the spiral microfluidic replica. For preliminary experiments on microparticle size selection, uncoated glass slides were used as the bottom substrates in the making of the spiral microfluidic chip. Untreated glass slides were cleaned with acetone and ethanol and dried under N_2_. Elsewhere, glass slides with PPOx coated reservoir were used to form the microfluidic chips. PPOx coated glass slides were used “as is” following plasma deposition, with no further cleaning. The PDMS cast was removed from the master, inlet and outlet holes were cut, and the PDMS and glass slides were plasma activated. Plasma activation was performed in a plasma cleaner (Harrick Plasma, United States). Samples were brought under vacuum (300 mTorr). Oxygen gas or atmospheric air was introduced to the chamber and the plasma was ignited at medium power once a working pressure of 800 mTorr was achieved. Samples were activated for 30 s, and immediately bonded together at the end of the cycle. The spirals formed have a total volume of 28.6, 39.1, and 49.4 µl for the 4, 8, and 12 loop designs respectively, with each reservoir bearing a volume of 8.6 µl.

### Spiral Microfluidics

Polystyrene microparticle solutions of 6, 10, and 20 µm purchased from Polysciences, were diluted to concentrations of 5.5 × 10^2^, 1.18 × 10^4^, and 5.5 × 10^2^ particles/mL respectively. 500 µl of particle solution was flowed into each microfluidic device at either 300 μl/min or 500 μl/min using a syringe pump (KD scientific). Outlet reservoirs were imaged using an Olympus IX83 microscope and particles were counted using CellSens Dimension v2.3.

### Selective Cell Capture

Two human cell lines, normal prostate epithelium PNT2 (# 95012613) and prostate carcinoma LNCaP clone FGC (# 89110211) were supplied by the European Collection of Cell Cultures (ECACC; Salisbury, United Kingdom), and were purchased from CellBank Australia (Westmead, NSW, Australia). RPMI-1640 media (Sigma Ladrich, NSW, Australia) was used to culture both PNT2 and LNCaP cells. The growth medium was supplemented with 10% fetal calf serum and 1% (v/v) penicillin/streptomycin (Thermofisher Scientific, Australia). Cells were cultured at 37°C in a humidified atmosphere containing 5% CO_2_.

Microfluidic chips containing six rectangular channel grooves were purchased from ibidi (Germany) and assembled onto a PPOx coated microscope slide following established protocol. A second PPOx coated slide was first activated by oxygen plasma before assembly. Two of the six channels in the ibidi slides were used as is, the biocompatible PPOx coating serving as a positive control surface onto which all cell types readily bind non-specifically. The next two channels were blocked with 50 µl of 1 mg/ml skim milk for 1 h at room temperature, and used as the negative control surface, the skim milk protein preventing the binding of all cell types. The last two test channels were functionalized with 70 µl of 10 μg/ml anti-PSMA antibodies overnight at 4°C before blocking with skim milk. All channels were rinsed with phosphate buffered saline (PBS) three times to remove unbound proteins. The outlet reservoirs of the spiral microchannels were functionalized in a similar manner, using separate devices for each condition (positive control, negative control and test). Through the outlets, 8.6 µl of 33.3 μg/ml anti-PSMA antibody solution was inserted into both the inner and outer reservoir and incubated overnight at 4°C. The next day, 1 mg/ml skim milk block was flowed from the inlet and incubated for 1 h before rinsing with 250 µl of PBS flowed at 300 μl/min.

LNCaP and PNT2 cells were stained using Nuclear-Red (0.5 μmol/L) (AAT Bioquest, United States) and NucBlue Live ReadyProbes Reagent (Hoechst 33342) (ThermoFisher Scientific, Australia) respectively for 10 min in separate tubes. The cell suspensions were centrifuged at 1,200 RPM for 5 min, the supernatant was removed and the pellets resuspended in fresh PBS before mixing the two cell types together in a 1:1 ratio. 50 µl of the mixed cell solution was added to each ibidi channel and 80 µl was flowed through each microfluidic device. The reservoirs were imaged upon introduction and settling of cells in microchannels, then left to incubate for 45 min. The ibidi channels were rinsed with 700 µl of PBS and the spirals were rinsed with 250 µl of PBS at 300 μl/min. The reservoirs were imaged after rinsing to determine the selective capture efficiency. Images were analyzed using cellSens software.

### PCR Analysis of the Captured Cells

#### RNA Extraction

Total RNA was extracted from the captured cells using the RNeasy Plus Micro Kit (Qiagen, VIC, Australia). 80 µl of RLT buffer was injected into the spiral inlet for captured cells lysis. The lysate was collected from inner and outer channels separately. Then, the RNA extraction was carried out as per the manufacturer’s instructions.

#### RT-PCR Validation

Extracted RNA was converted to cDNA using QuantiTect Reverse Transcription Kit (Qiagen, VIC, Australia) following the manufacturer’s protocols. The cDNA was then preamplified using TaqMan PreAmp Master Mix Kit with targeted TaqMan Gene Expression Assays (Thermo Fisher Scientific, VIC, Australia). Targeted genes assays used included androgen receptor (AR) (Hs00171172_m1), kallikrein related peptidase 3 (KLK3) (Hs02576345_m1), prostate cancer associated 3 (PCA3) (Hs01371939_g1), prostate-specific membrane antigen (PSMA) (Hs00379515_m1) and glyceraldehyde-3-phosphate dehydrogenase (GAPDH) (Hs02786624_g1). Real-time RT-PCR was performed on LightCycler 480 Instrument II (Roche Diagnostics, NSW, Australia). The PCR reaction was set in 10 µl sample, which contains 2 µl of preamplified and diluted cDNA, 2× TaqMan Universal Master Mix II with UNG and each 20× primer/probe. The RT-PCR was performed using the following cycling condition: 2 min at 50°C, 10 min at 95°C for polymerase activation, and 50 cycles of 15 s at 95°C and 1 min at 60°C. For each gene, triplicate PCR were performed.

## Results

### PPOx Characterisation Following Plasma Activation

MePOx and PiPOx films were deposited for 30 and 50 s with a plasma ignition power of 20 W and a monomer working pressure of 1.2 × 10^−1^ mbar, according to established protocols (Chan K. M. et al., 2020). The thickness of the untreated films ranged from 6.6 to 11.5 nm, increasing with both deposition time and monomer size.

These results are in good agreement with previous reports and indicate that the films are deposited in a monomer sufficient regime where film growth mechanisms are dominant over concurrent etching effects (Macgregor-Ramiasa et al., 2015). Following air plasma treatment, all the PPOx films investigated suffered a 2.5 nm thickness loss, irrespective of the type of monomer used or deposition time. In contrast, thickness loss was markedly more pronounced for PiPOx than for MePOx following oxygen plasma treatment. Plasma activation works for the bonding of PDMS devices by etching contaminants and the top atomic layer(s) of the substrates away while creating oxygen containing silanol groups on the surface. Pure oxygen plasma treatment is more effective than air plasma treatment because reactive oxygen species are more energetic than reactive nitrogen species. The thickness loss observed in PPOx films results from these etching mechanisms, which are more pronounced on soft organic layers than on glass. Structural differences between MePOx and PiPOx films could explain the greater thickness loss experienced by PiPOx following the harsher O_2_ plasma treatment. Indeed, previous studies have indicated that plasma polymers deposited from unsaturated monomers displayed lower elastic modulus than their saturated analogues (Michelmore et al., 2013).

**FIGURE 3:**
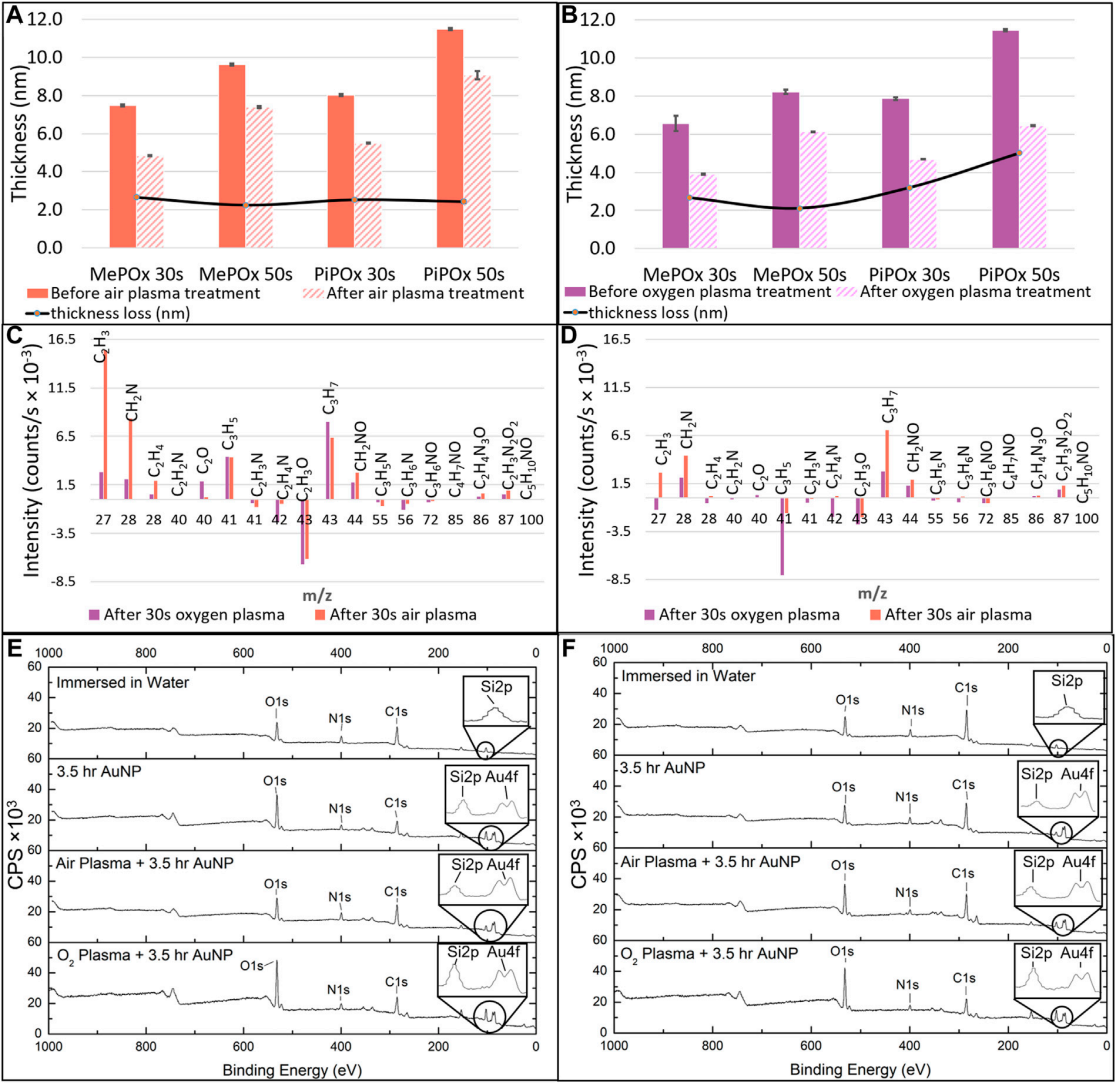
MePOx and PiPOx film thickness as determined by ellipsometry before and after 30 s air plasma treatment **(A)** and oxygen plasma treatment **(B)**. Relative changes in molecular fragments detected by ToF-SIMS before and after oxygen or air plasma treatment on MePOx films **(C)** and PiPOx films **(D)**. XPS survey spectra for MePOx **(E)** and PiPOx **(F)** 50 s films after 3.5 h immersion in water, and after 3.5 h incubation with AuNPs with and without prior air and oxygen plasma activation. CPS refers to counts per second.

Next the changes occurring in the films surface chemistry following plasma activation were investigated. Figure 3 presents the results of TOF-SIMS analysis conducted on MePOx and PiPOx films in terms of relative changes for molecular fragments with m/z < 100 following oxygen and air plasma activation. Both PPOx films have relatively higher amounts of aliphatic fragments C_2_H_3_ and C_3_H_7_ after plasma activation and relatively lower amounts of heteroatomic fragments C_2_H_4_N and C_2_H_3_O. This is in good agreement with the expectation that oxygen and air plasma species react with heteroatoms present in the film. The main difference between MePOx and PiPOx is the relative change in the C_3_H_5_ fragment, which corresponds to the pendent group of PiPOx. The fact that this fragment decreases for PiPOx post plasma activation could indicate that intact isopropenyl groups present on the PiPOx surface post plasma deposition were subsequently etched off by the oxygen reactive species (more so than by the air plasma). It is also worth noting that the fragments with m/z 72, 85, and 86, which correspond to the oxazoline ring itself, did not vary following plasma activation. This indicates that this functionality, just like the other fragments containing both nitrogen and oxygen, is not affected by oxygen or air plasma. Overall, for both MePOx and PiPOx, greater changes occurred in the distribution of the molecular fragments following air plasma treatment. In order to determine whether this observation translated to corresponding changes in the reactivity of the POx films, gold nanoparticles functionalised with mercaptosuccinic acid were left to incubate on the POx films as a model COOH-bearing ligand and investigated via XPS.

XPS analysis indicates that for both MePOx and PiPOx, the nitrogen content of the films was higher following air than O_2_ plasma treatment (Supplementary Table SI1). This could be due to the air plasma ablating less nitrogen containing species from the surface. Or it could be due to reactive nitrogen species generated in the air plasma reacting with the PPOx films to form new additional nitrogen containing functions. In either case, the higher nitrogen content also correlated with higher reactivity toward COOH-AuNPs. In fact, following oxygen plasma treatment, AuNPs did not bind to the thinner MePOx and PiPOx films (30s deposition, Table 1). These thin films did not perform well following air plasma either as only residual amounts of AuNPs bound to MePOx, and PiPOx lost 20% of its binding capacity. These results are in good agreement with those of the TOF SIMS, in showing that the surface chemistry of MePOx is less stable than that of PiPOx. In contrast, the thicker films (deposited for 50s) retain good reactivity toward the COOH-AuNPs. The differences in the amount of gold measured on the surface following plasma treatment were not statistically significant amongst the 50s films (Table 1), indicating that sufficient film thickness (above 8 nm) is the most critical parameter.

**TABLE 1:** Atomic percentage of gold detected on MePOx and PiPOx surfaces after 3.5 h incubation with 68 nm AuNP solution, before and after 30 s of oxygen or air plasma treatment.

% AuNP	MePOx, 30 s	MePOx, 50 s	PiPOx, 30 s	PiPOx, 50 s
Untreated POx	2.4 ± 0.03	2.3 ± 0.02	2.4 ± 0.09	2.2 ± 0.1
Air plasma	0.05 ± 0.07	2.2 ± 0.1	1.9 ± 0.2	2.2 ± 0.5
Oxygen plasma	0 ± 0	1.9 ± 0.5	0 ± 0	2.0 ± 0.5

### Spiral Microfluidics for Size Selection

Having shown that the PPOx could undergo plasma activation without losing its reactivity, the next step was to integrate these films into a spiral microfluidic chip for cell isolation and preconcentration. The spiral microfluidic design was first optimised to induce the separation of microparticle with size comparable to that of cells found in body fluid, from 20 µm (e.g., epithelial cells), down to 6 µm (e.g., red blood cells). Particles flowing in curved microchannels are acted upon by three principal forces: a wall induced lift force (F_WL_) directs the particles toward the channel midline, a shear gradient lift force (F_SL_) toward the channel wall, and a secondary drag from Dean vortices arising from the fluid’s momentum from centrifugal acceleration (F_D_) (Zhang et al., 2016; Liu et al., 2019). Parametric analysis of these forces mathematical expression (provided in Supplementary Material) indicate that particle size, spiral radius of curvature and fluid velocity (flow rate) are the most important variables in particle focusing in a spiral microchannel. Therefore the separation efficiency of the different sized particles were investigated in spirals with increasing numbers of loop at different flow rates.

**FIGURE 4:**
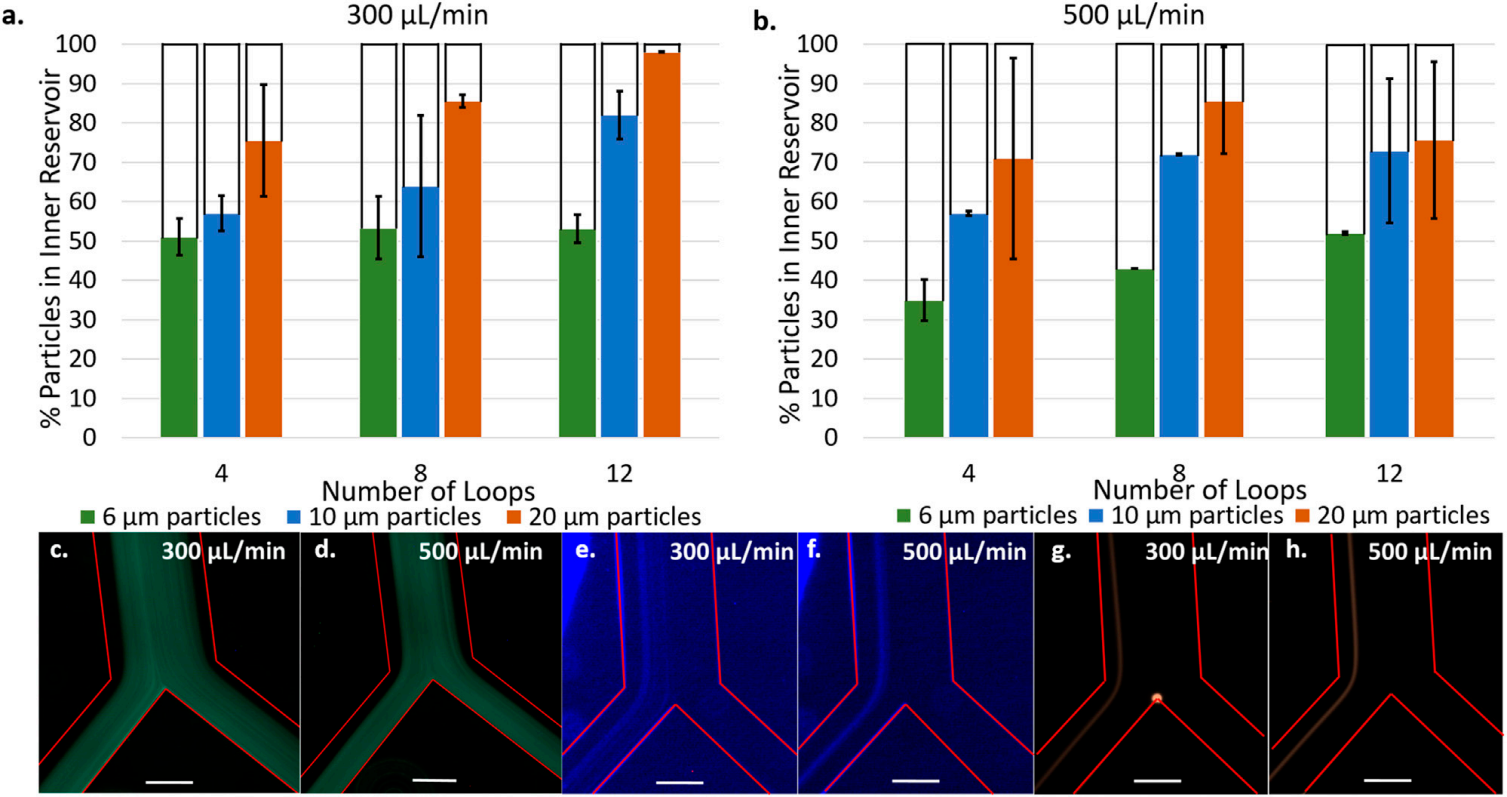
Percentage of particles counted in inner reservoir in devices with 4, 8, and 12 loop spirals with 6, 10, and 20 µm particles at 300 μl/min **(A)** and 500 μl/min **(B)**. [6 µm particles] = 5.5 × 10^2^ particles/mL, [10 µm particles] = 1.18 × 10^4^ particles/mL, [20 µm particles] = 1.48 × 10^4^ particles/mL. Fluorescence microscopy images at bifurcation of 6 µm particles flowing in 12 loop spirals at 300 μl/min **(C)** and 500 μl/min **(D)**. Fluorescence microscopy images at bifurcation of 10 µm flowing in 12 loop spirals at 300 μl/min **(E)** and 500 μl/min **(F)**. Fluorescence microscopy images at bifurcation of 20 µm flowing in 12 loop spirals at 300 μl/min **(G)** and 500 μl/min **(H)**. Red lines delineate microchannel walls, scale bar represents 250 μm [6 µm particles] = 2.18 × 10^5^ particles/mL, [10 µm particles] = 4.73 × 10^4^ particles/mL, [20 µm particles] = 1.48 × 10^4^ particles/mL.

Figures 4A,B present the separation efficiency of 6, 10, and 20 μm fluorescent polystyrene particles at flow rates of 300 μl/min a) and 500 μl/min b) in spirals consisting of 4, 8, and 12 loops. Spirals of 2, 6, and 10 loops were also tested and the data is presented in Supplementary Figure SI2. At both flow rates, increasing the number of spiral loops increases the separation efficiency of 10 and 20 μm particles, up to a maximum of 82 and 98% at 300 μl/min in the 12 loop spirals. It was not the case with 6 μm at 300 μl/min and only a small improvement is observed at 500 μl/min for this size of microparticles. These results are in good agreement with equations 1, 2, and 4 (Supplementary Material) which predict that larger particles will experience greater shear gradient lift force and therefore focus closer to the inner wall than smaller ones.

Fluorescent micrographs of the particle focusing band at the bifurcation (Figures 4C–H) provides further insight into the particle positions across the microchannel cross section. Figures 4E,F show 10 μm particles focusing in a narrow band centered around 140 μm from the channel inner wall with a width of 136 μm at 300 μl/min and 97 μm at 500 μl/min (see line scans Supplementary Figure SI3). There is no significant change in the position of the focused band at different flow rates, highlighting the dominance that particle size has on the equilibration point. However, the increased flow rate does narrow the focusing band. Figure 4G,H present 20 μm particles focusing in narrow bands centered around 103 μm from the channel inner wall with a width of 68 μm at 300 μl/min and centered around 86 μm from the inner wall at 500 μl/min with a width of 57 μm at 500 μl/min. As expected, 20 μm particles focus closer to the inner wall and in a narrower band than 10 μm particles. Unlike the 10 μm particles, the position of equilibrium for these particles occurred closer to the channel inner wall when the flow rate was increased (Supplementary Figure SI3). It is possible this occurred due to the larger size being more sensitive to changes in flow rate. These observations are consistent with the equations presented in the Supplementary Material as the lift forces depend on the channel Reynolds number, $Re_{c}$, which is directly proportional to flow rate (equation 3).

Although we observe a narrower focusing band at 500 μl/min, higher separation efficiency was achieved at 300 μl/min. This is attributed to initial turbulences arising upon introducing the particle suspension into the spiral at the highest flow rate. Indeed, at 500 μl/min, chaotic mixing was observed at the inlet. The initial chaotic mixing may prevent particles present in the first 10–20 μl of solution from focusing into an equilibrium band as observed in Figures 4E–H, contributing to the lower separation efficiency and replicability at 500 μl/min. Figures 4C,D show 6 μm particles are focused on the channel midline and are split evenly into the two outlets, resulting in the observed 50% separation efficiency independent of flow rate. Similarly to 10 and 20 μm particles, increasing the flow rate narrows the focusing band (Supplementary Figure SI3 – line scans) but these particles are too small to be affected by the lift forces being exerted. Therefore, 12 loop spirals at a flow rate of 300 μl/min are proposed as the conditions where optimal separation efficiency is observed. This is also supported by Supplementary Figure SI4 presenting the separation of LNCaP cells being most efficient at these conditions compared to 4, 6, and 10 loop spirals.

### Size Separation in POx Coated Sprials

**FIGURE 5:**
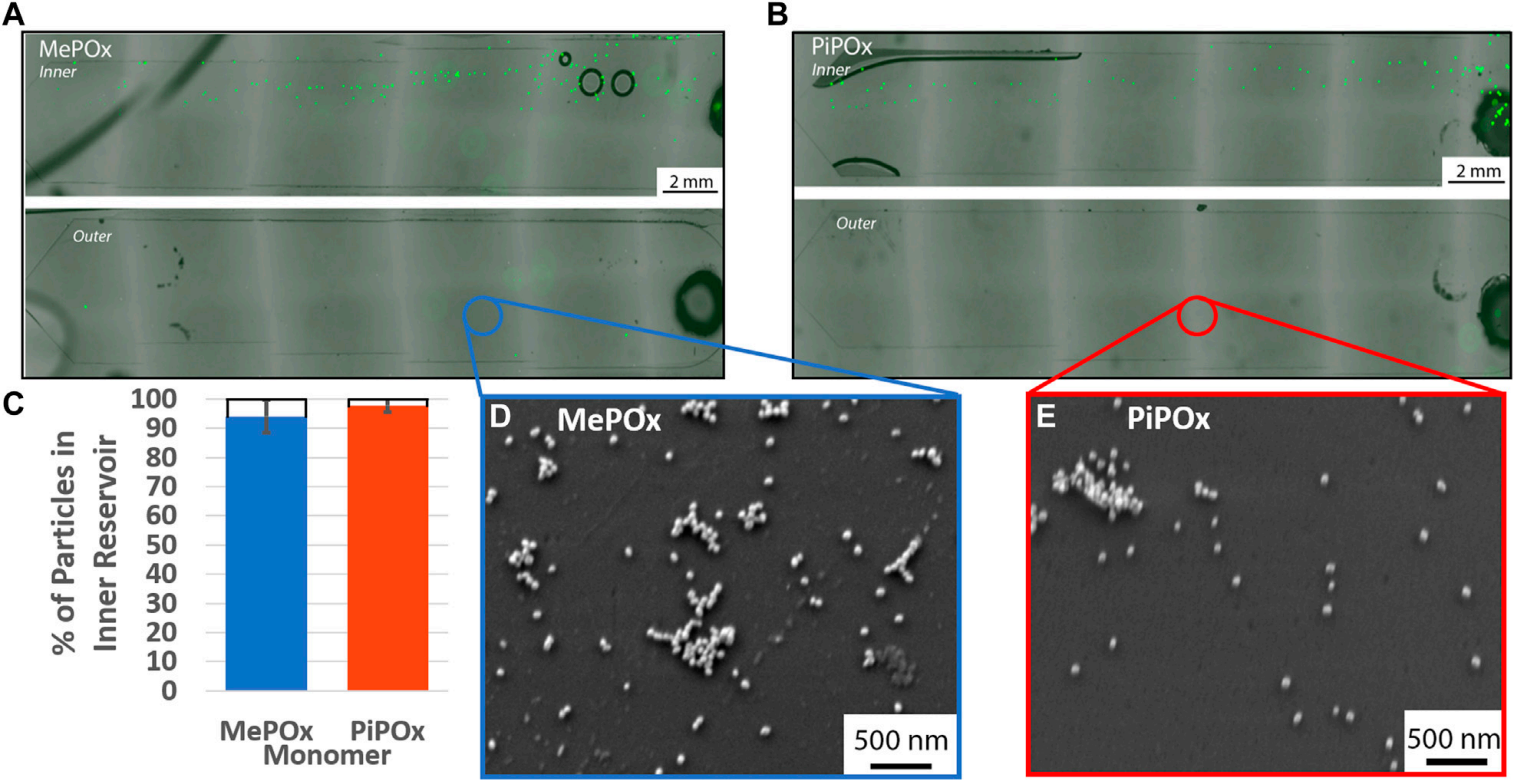
Representative fluorescent images of inner **(top)** and outer **(bottom)** reservoirs functionalised with MePOx **(A)** and PiPOx **(B)** containing separated 20 µm particles from a 1:9 mixture of fluorescent particles and 68 nm AuNPs. **(C)** Separation efficiency of 20 µm particles in a 1:9 mixture of fluorescent particles and 68 nm AuNPs. SEM images of the reservoir surface showing AuNP captured on MePOx **(D)** and PiPOx **(E)** from the particle mixture. Separation conditions used: 12 loop spiral, 300 μL/min.

The separation efficiency of the 12 loop spiral was then tested in the presence of PPOx coated reservoirs. In these tests, various concentrations of 20 µm microparticles were mixed with 68 nm nanoparticles, to evaluate the influences of particle-particle interaction on the separation efficiency. The nanoparticles served as a model for exosomes, which may also become the target of biosensing applications of the device. The size separation of the 20 µm microparticles remain unaffected by the presence of nanoparticles in either MePOx and PiPOx coated spirals, Figures 5A–C. In both cases, more than 90% of the microparticles were directed into the inner reservoir. Particle-particle interactions can have an effect on particle focusing through hydrodynamic shear-induced diffusion (equation 6, Supplementary Material). Callens et al. (2008) However, this parameter is proportional to the particle radius squared, thus the AuNPs present in this mixture do not significantly affect the microparticles migration.

According to force calculations, nanoparticles are too small for their trajectory to be influenced by the centrifugal forces acting in the spirals. Our results confirm these theories as nanoparticles were present in both the inner and outer reservoirs. Furthermore, the AuNPs bound to the PPOx coatings, Figures 5D,E, indicating that the reactivity of the PPOx was maintained within the microfluidic chip despite plasma activation, and that the high shear rates did not disrupt their bond.

Not only were the 20 µm particles directed toward the inner reservoir, they were in fact distributed toward the inner edge of the reservoir itself. This indicates that the microparticles focused toward the inner wall of the inner bifurcation channel, which means that the bifurcation could potentially be more restrictive and continue to capture the vast majority of 20 µm microparticle. Altering the bifurcation design to direct more than 50% of the fluid to the outer outlet could be beneficial in the capture of exosomes, which are too small to be focused by this centrifugal force. A stream thinner than 50% of the channel width could be used to separate the cells on the inner side, keeping a large proportion of the “cell-free supernatant” available for urinary exosomes interrogation in the outer reservoir.

### Integration of the PPOx Into the Spiral

Cell suspensions containing a 1:1 mixture of healthy and prostate cancer cells were injected into the microspirals at a flow rate of 300 μl/min. The cells were then allowed to settle in the capture reservoir and micrograph images were taken. This first set of images were used to determine the initial number of cells. After 45 min of incubation, the spirals were flushed with PBS to dislodge loosely bound cells and additional micrograph images of the captured cells were recorded to determine the capture efficiency. Following the same procedures, cell capture efficiencies were also determined for straight Ibidi channels as a control. For one set of Ibidi channels, the PPOx coating was used as is, whereas another set of PPOx was subjected to 30 s oxygen plasma activation prior to the biofunctionalization step. The result of the cell capture in the straight Ibidi channels controls, provided in Supplementary Figure SI5, show that more than 85% of cancer cells against less than 10% of healthy cells were captured in the anti-PSMA functionalised channel, whether or not the PPOx had undergone plasma activation. These results demonstrate that the reactivity of POx toward PSMA antibodies was retained following plasma activation.

**FIGURE 6:**
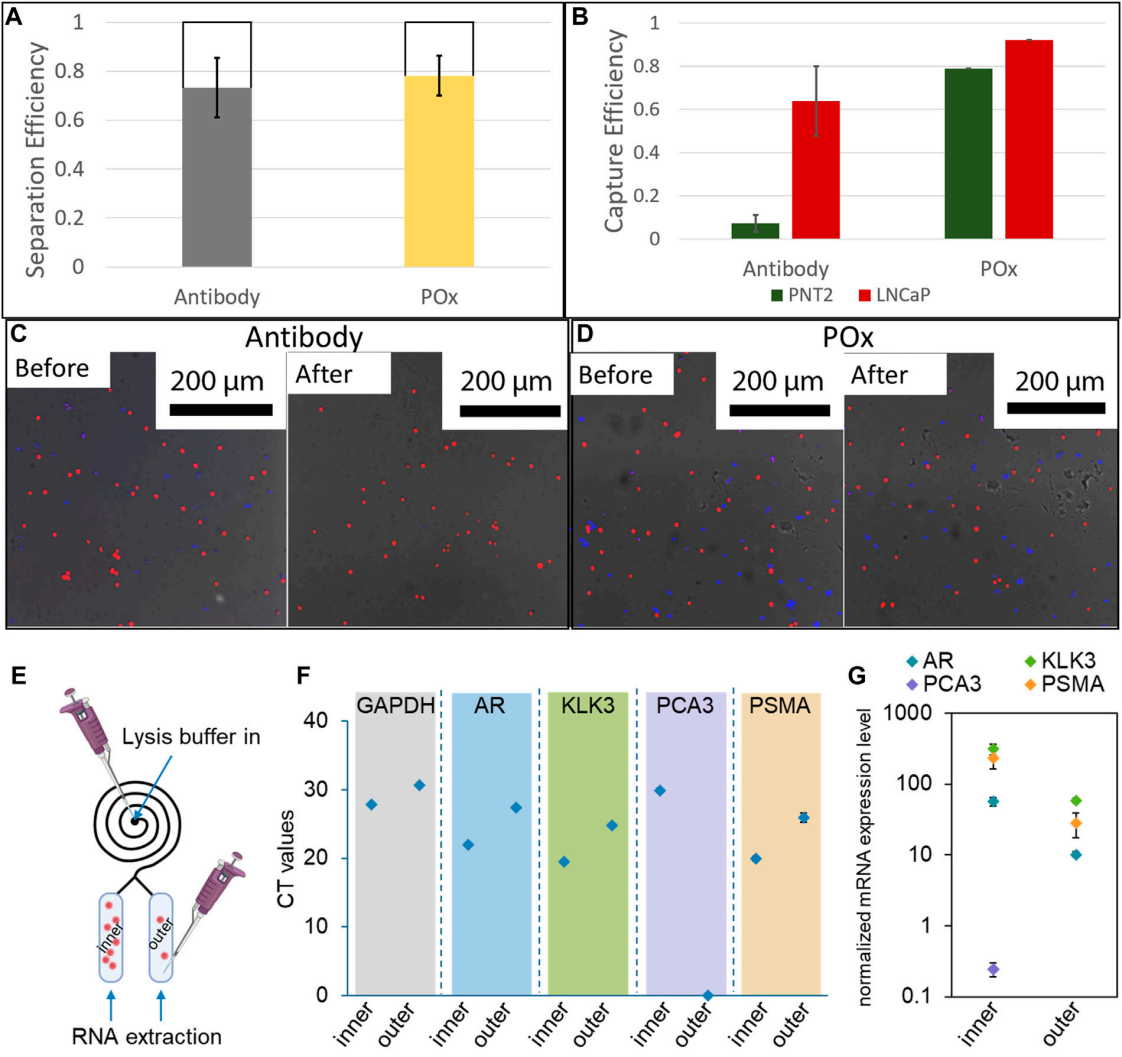
**(A)** Cell separation efficiency in 12 loop spirals at 300 μl/min (n = 3). **(B)** Selective capture efficiency of a mixture of LNCaP and PNT2 cells in anti-PSMA functionalised channels with 1 mg/ml skim milk block and unfunctionalised POx. Fluorescence micrographs of capture surfaces before and after PBS rinse on antibody functionalised POx **(C)** and unfunctionalised POx **(D)** (n = 3). A schematic illustration showing the captured cells lysis for RNA extraction **(E)**; results of real-time RT-PCR showing the **(F)** CT values and **(G)** expression levels of targeted genes after normalized with GAPDH (n = 3).

The result of the cell size selection from the spiral are shown in Figure 6A. For both anti-PSMA and PPOx channel, the vast majority of cells are found in the inner reservoir, though the separation was less selective (between 65 and 79%) than for 20 µm microparticles. This could result from the cell diameter ranging between 10 and 20 µm and that their density differs to that of the polystyrene microparticles. Nonetheless, following the PBS rinse, selective capture of cancer cells was achieved in the anti-PSMA functionalised inner channel. In this case 70% of the cancer cells and less than 7% of the healthy cells were retained. The lower capture efficiency could be imparted to the differences in geometry of the reservoir compared to the straight ibidi channel. Indeed, the reservoirs are slightly thinner and half the height. As a result, the sheer stress acting on the surface bound cells during the rinsing step are larger in the reservoir than they are in the Ibidi channels. It is also worth noting that degassing occurred in the spiral microfluidic which resulted in the formation of bubbles in the reservoir, potentially contributing to the lower measured capture rates. Optimising the rinsing flow rate and addressing degassing issues will constitute the object of future works.

At last, proof of concept PCR analysis was conducted on the cell captured in both the inner and the outer reservoir functionalised with anti-PSMA. Lysis buffer was introduced in the spiral, and the RNA was subsequently extracted through the outlets for each reservoir independently. The expression of prostate cells and prostate cancer specific mRNA was assessed. In both cases, and despite the low number of cells present in the outer channel, prostate specific biomarker expression was detected. Prostate cancer specific biomarker PSMA and PCA3 expression was also detected from the cellular material extracted from the inner channel. In the outer channel, the expression level of these prostate cancer markers was relatively low, in good agreement with the very low number of cells captured. Using calibration curves established with known numbers of LNCaP cells, the CT values were used to estimate the number of cells present in each reservoir, Supplementary Figure SI6. For all biomarkers investigated, the estimated cell numbers were one order of magnitude higher in the inner channel compared to the outer channel, in good agreement with the actual number of cells recorded on the micrographs. These results show that the microfluidic spiral could be used to enrich the population of cancer cells for both fluorescence microscopy and PCR analysis. One potential application of this system could be the side by side analysis of cellular and exosomic content of urine samples of interest, using microscopy and genomic analysis in the inner and outer channels respectively.

## Conclusion

This work demonstrates that nanothin PPOx films can be integrated into plasma bonded microfluidic chips without compromising reactivity toward COOH-bearing ligands. The thickness of the films is a critical parameter in retaining sufficient film reactivity. Specifically, methyl and isopropenyl films thicker than 8 nm readily bind COOH-AuNPs following a 30 s air or oxygen plasma activation. The PPOx films can be integrated into reservoirs at the exit of microfluidic chips designed for the size-specific isolation of microparticles. Particle-particle interactions did not affect the size separation potential in 12 loop spirals, and COOH-AuNPs nanoparticles were captured on the PPOx surface upon exiting the spiral, indicating that the system could potentially be used for the detection of nano-sized biomarkers such as exosomes. In mixed cell suspensions, the immunofunctionalised PPOx surface enables the selective capture of prostate cancer cells based on their over-expression of PSMA.

## Data Availability

The raw data supporting the conclusions of this article will be made available by the authors, without undue reservation.
