# Supplementary material for: Plasma Deposited Polyoxazoline Films Integration Into Spiral Microfluidics for the Targeted Capture of Size Selected Cells
Source: Front Chem. 2021 May 20;9:690781. doi: 10.3389/fchem.2021.690781 (PMC8172585; doi:10.3389/fchem.2021.690781)
Supplement: Supplementary file 1 [file DataSheet1.PDF]

Plasma deposited polyoxazoline films integration into spiral microfluidics for the targeted capture of size selected cells

Alexandru A. Gheorghiu[1], Ines Muguet[2], James Chakiris[1], Kit Man Chan[1], Craig Priest[1], Melanie Macgregor[1*]

[1] Future Industries Institute, University of South Australia, Mawson lakes, SA 5095, Australia
[2] École Nationale Supérieure de Chimie, de Biologie et de Phisique de Bordeaux, Bordeaux INP, 33607 Pessac, France

*** Correspondence:**
Melanie MacGregor
melanie.macgregor@unisa.edu.au

Supplementary Information

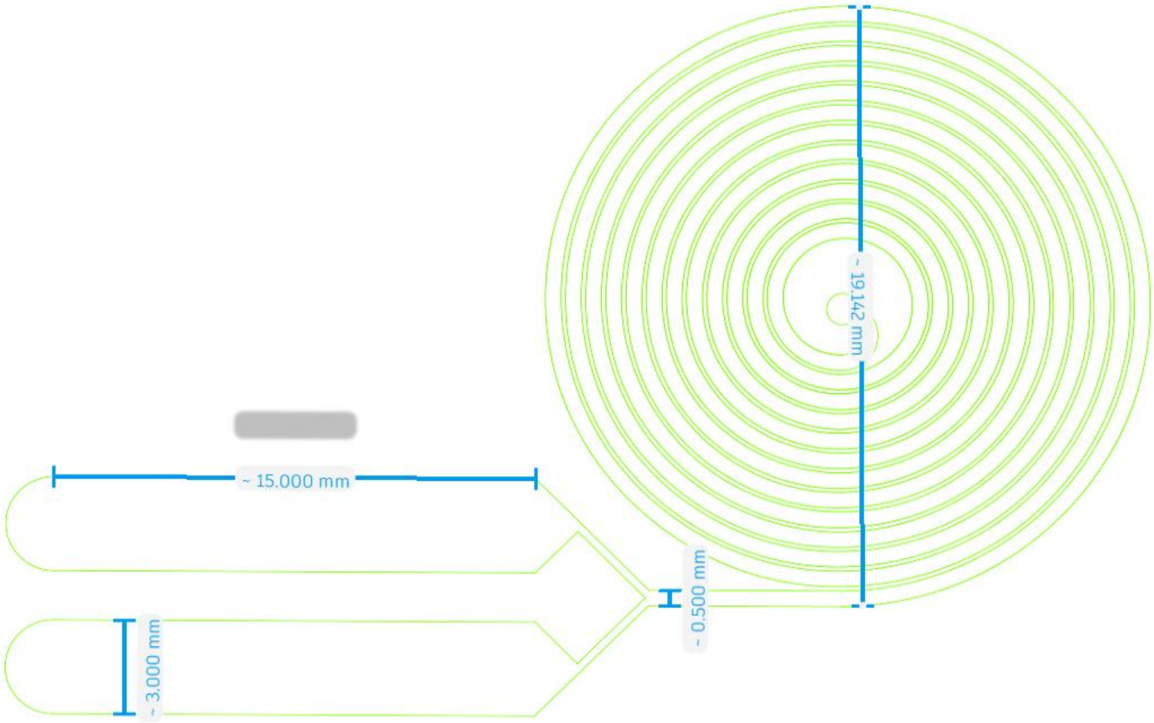

Figure SI1: CAD drawing of a 12 loop spiral with reservoirs and measurements of channel dimensions

Table S1: Percentage of atomic Nitrogen detected on films after 3.5 hr immersion in water, after 3.5 hr AuNP incubation, after AuNP incubation post air plasma activation, and after AuNP incubation post O_2 plasma activation

% Nitrogen	MePOx 30s	MePOx 50s	PiPOx 30s	PiPOx 50s
**Immersed in Water**	7.81 ± 0.6	10.1 ± 0.5	7.36 ± 0.8	8.19 ± 0.05
**3.5 hr AuNP Incubation**	6.38 ± 0.005	7.42 ± 1.4	5.66 ± 0.7	8.31 ± 0.4
**Air Plasma + 3.5 hr AuNP Incubation**	3.15 ± 0.2	9.58 ± 0.005	2.82 ± 0.3	6.16 ± 0.7
**O_2 Plasma + 3.5 hr AuNP Incubation**	0.00 ± 0	3.01 ± 0.5	0.00 ± 0	4.88 ± 0.3

Mathematical expressions of the forces acting on the particles in the spiral microfluidics :

Wall induced lift force:

$$F_{WL} = \frac{\rho U_m^2 a^6}{D_h^4} f_{WL}(Re_c, x) \ \dots (1)$$

Shear gradient lift force

$$F_{SL} = \frac{\rho U_m^2 a^3}{D_h} f_{SL}(Re_c, x) \dots (2)$$

Channel's Reynolds numbers

$$Re_c = \frac{\rho U_m D_h}{\mu} \dots (3)$$

Decondary Dean drag force

$$F_D = 5.4 \times 10^{-4} \pi \mu De^{1.63} a \dots (4)$$

$$De = Re_c \sqrt{\frac{D_h}{2R}} \ \dots (5)$$

Where ρ is the fluid density, U_m is the maximum velocity of fluid flow, a is the particle size, $D_h = \frac{2wh}{w+h}$: the hydraulic diameter for a rectangular channel, and $f(Re_c, x)$ is the lift coefficient that depends on the channel Reynolds number (Re_c) and the particle's position in the channel (x), μ is the dynamic viscosity of the fluid, R is the spiral's average radius of curvature, and De is the Dean number. The Dean number is a dimensionless value describing the dean drag.

Hydrodynamic shear induced diffusion:

$$D_h = c\dot{\gamma} a^2 h(\varphi)$$

Where c is a constant, $\dot{\gamma}$ is the shear rate, and h(φ) is an increasing function of the particle volume fraction φ.

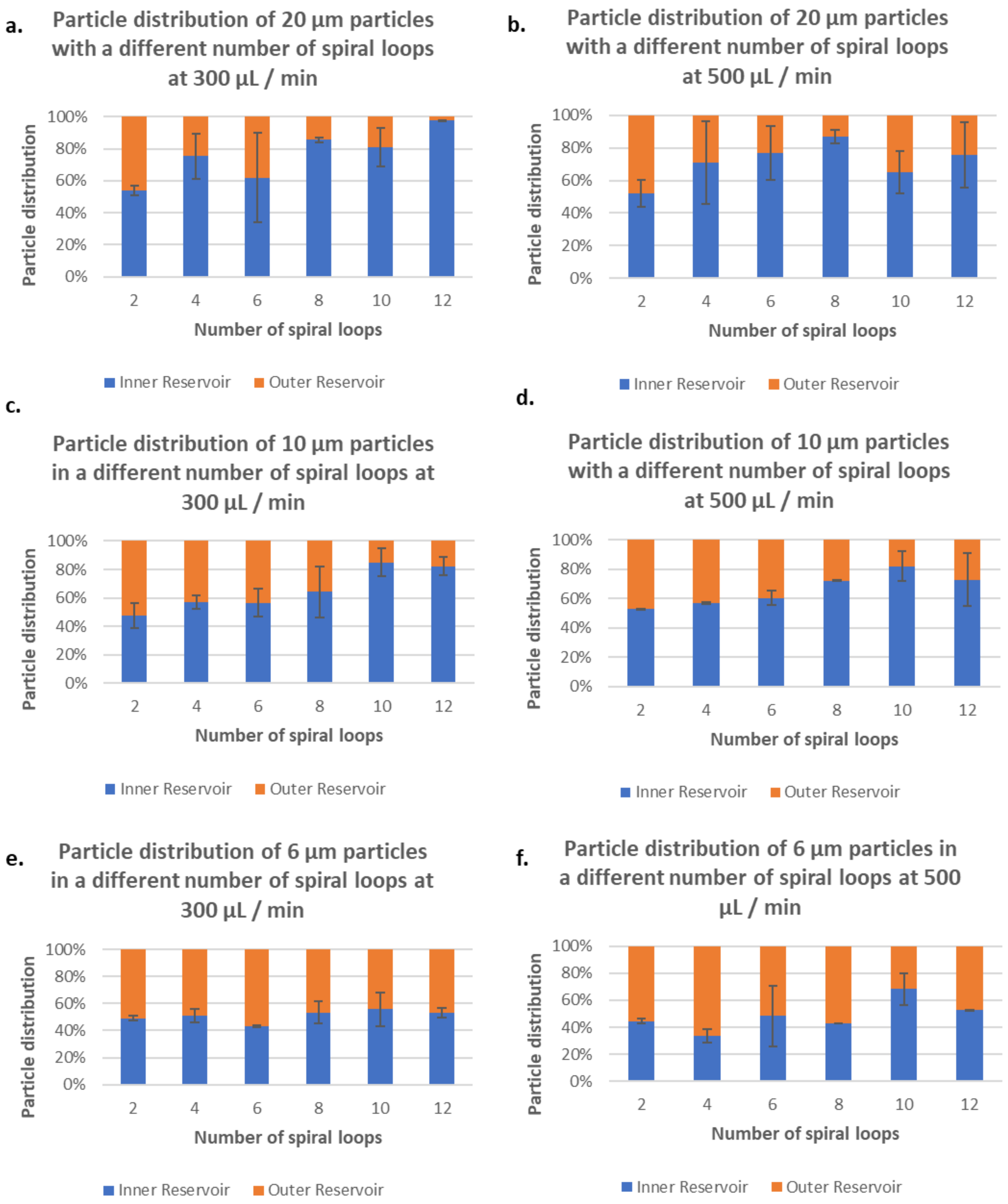

Figure SI2: Particle distributions of 6, 10, and 20 µm particles with a different number of spiral loops at 300 µL/min and 500 µL/min

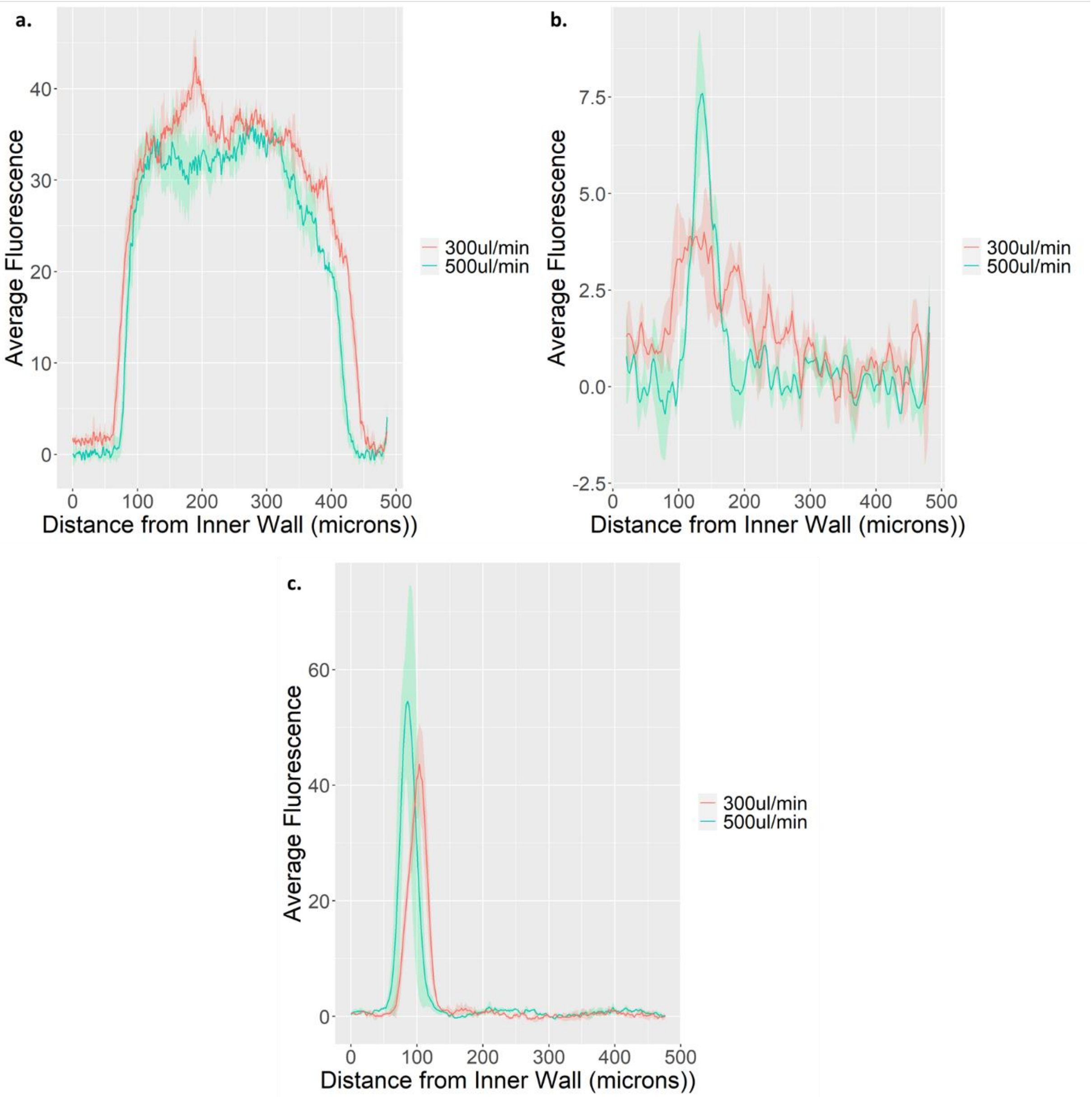

Figure SI3: Line scan traces across the channel at the bifurcation of focussed 6 micron particles (a), 10 micron particles (b), and 20 micron particles (c) at 300 µL/min and 500 µL/min. Solid line represents average and shaded area represents standard deviation. Background subtraction was performed on each data set.

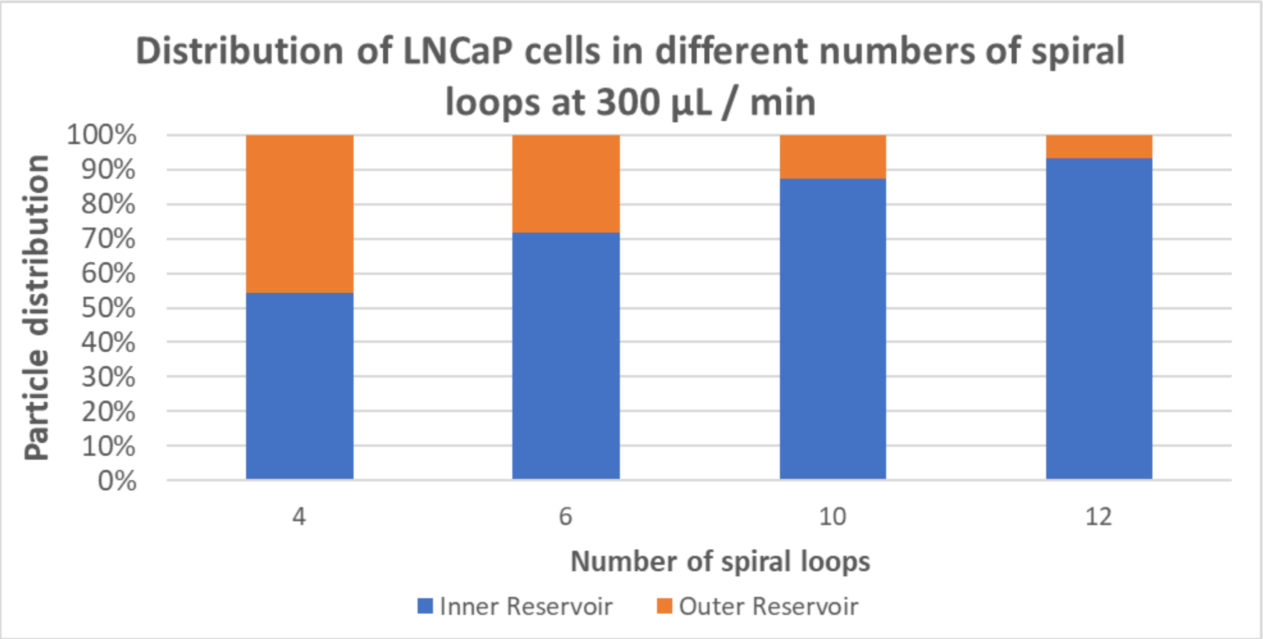

Figure SI4: Distribution of LnCAP cells in devices of increasing loop numbers at 300 µL/min. Cell concentration of 4.8 x 10^4 cells/mL.

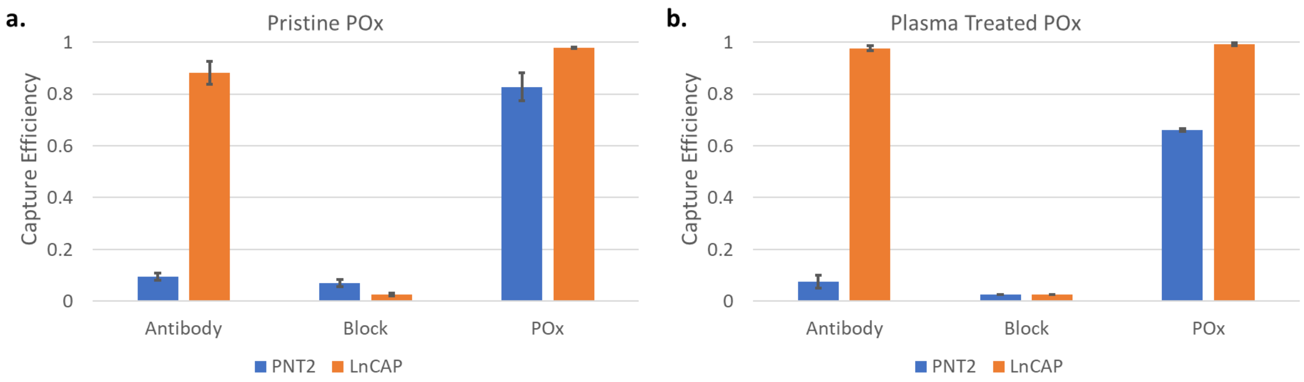

Figure SI5: Selective cell capture efficiency of LnCAP and PNT2 cells in ibidi channels on pristine POx (a) and O_2 plasma treated POx (b).

a

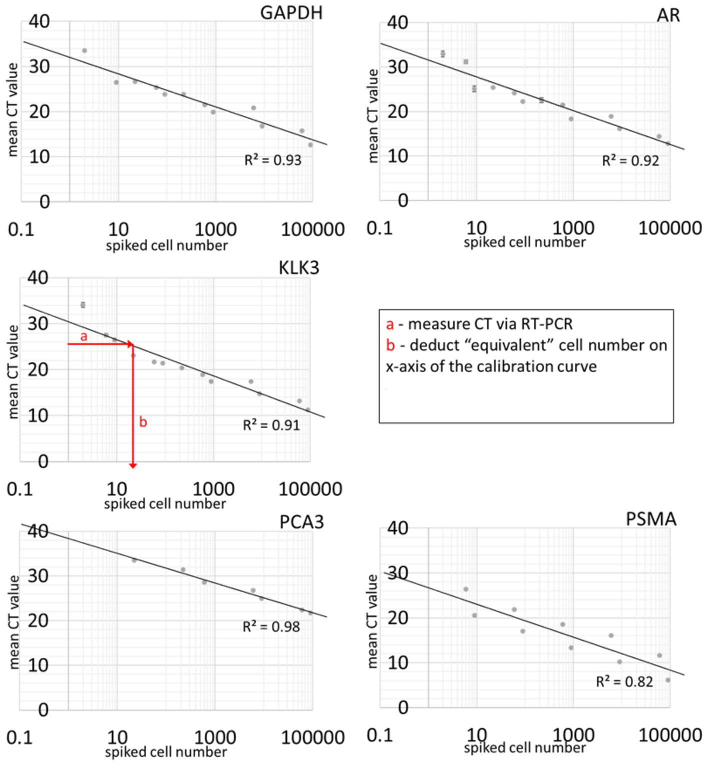

b

	cell no. corresponding to calibration curve				
	GAPDH	AR	KLK3	PCA3	PSMA
Test inner	16	300	600	540	68
Test outer	3.1	13	30	0	1.6
POx inner	<0.01	13	1.6	19	<0.1
POx outer	0.13	120	72	0	12

c

	CT				
	GAPDH	AR	KLK3	PCA3	PSMA
Test inner	27.80	21.99	19.54	29.86	19.97
Test outer	30.68	27.34	24.81	0.00	25.93
POx inner	43.09	27.22	29.86	35.34	31.52
POx outer	35.41	23.77	23.03	0.00	22.81

d

	2^(-ΔCt)			
	AR	KLK3	PCA3	PSMA
Test inner	56.21	307.88	0.24	227.72
Test outer	10.13	58.21	0.00	26.84
POx inner	59999.46	9610.99	215.18	3031.09
POx outer	3173.87	5311.25	0.00	6182.74

Figure SI6: (a) spiked-in calibration curves used to estimate the cell number in samples; (b) estimated number of cell/ml in channels according to the calibration curves; (c) and (d) RT-PCR validation results.
